# Do Multimodal Vision-Language Models Enhance the Medical Diagnostic Process? A Systematic Review

**DOI:** 10.3390/healthcare14131877

**Published:** 2026-06-26

**Authors:** Lattawat Eauchai, Laura Otálora González, Yifan Shi, Michele T. McGinnis, Alexander Yovchev, Svetlana Herasevich, Brian W. Pickering, Vitaly Herasevich

**Affiliations:** 1 Department of Anesthesiology and Perioperative Medicine, Division of Critical Care, Mayo Clinic, Rochester, MN 55905, USA; eauchai.lattawat@mayo.edu (L.E.); otaloragonzalez.laura@mayo.edu (L.O.G.); shi.yifan@mayo.edu (Y.S.); yovchev.alexander@mayo.edu (A.Y.); herasevich.svetlana@mayo.edu (S.H.); pickering.brian@mayo.edu (B.W.P.); 2 Mayo Clinic Libraries, Mayo Clinic, Rochester, MN 55905, USA; mcginnis.michele@mayo.edu

**Keywords:** vision-language model, VLM, patient history, medical history, diagnostic performance, image, systematic review

## Abstract

**Highlights:**

**What are the main findings?**

- Multimodal diagnostic vision-language models (VLMs) consistently outperformed unimodal models using either text or image data alone.
- Physicians with VLM assistance achived higher diagnostic accuracy compared to physicians without VLM support. However, there is conflicting evidence for VLMs as standalone diagnostic agents.

**What are the implications of the main findings?**

- The consistent superiority of multimodal VLMs implies that future diagnostic systems require architectures that can effectively process imaging and clinical text to capture the necessary context for accurate diagnosis.
- Given the conflicting evidence for standalone AI versus the copilot model, implementation should focus on AI-assisted workflows that augment human expertise rather than replacing it, positioning VLMs as robust clinical decision support tools to enhance workflow, mitigate physician fatigue, and reduce cognitive bias.

**Abstract:**

**Background/Objectives**: Novel vision-language models (VLMs) can integrate patient textual data with image data to support medical diagnosis. Recent studies reported conflicting results regarding the performance of multimodal VLMs compared to other models and physician performance. This systematic review aims to assess the diagnostic performance of multimodal VLMs integrating both patient textual and image data across diverse real-world hospital settings. **Methods**: We performed comprehensive searches of eight resources, including Embase, MEDLINE, and SCOPUS, on 17 December 2025. Eligible studies reporting diagnostic performance of VLMs integrating both image and patient history textual data from real-world adult patients compared to that of other models and physicians were included. The review adhered to the Preferred Reporting Items for Systematic Reviews and Meta-Analyses (PRISMA) guidelines. The Prediction model study Risk Of Bias Assessment Tool + AI (PROBAST + AI) was used to assess the quality and risk of bias. The study protocol was registered in the PROSPERO database (CRD420251244054). This review received no external funding. **Results**: We screened 11,026 records, of which 18 studies met the inclusion criteria. Six studies comparing multimodal and unimodal models demonstrated the consistent superiority of the multimodal models. Four studies evaluating VLM accuracy as standalone agents compared with physician performance reported conflicting evidence. One study assessing VLMs as a clinical copilot demonstrated higher accuracy from the group of physicians using VLM assistance. A meta-analysis could not be performed due to the heterogeneity across study populations and outcomes. The majority of the studies were assessed as having a high risk of bias due to dataset quality. Primary limitations identified across studies include small sample size, a lack of external validation, and the need for prospective clinical deployment studies. No study provided documented considerations regarding model safety or data security. **Conclusions**: This systematic review suggests that multimodal VLMs consistently outperform unimodal models with access to only image or text. While model performance as standalone agents compared to humans remains inconclusive, a copilot model has demonstrated high diagnostic accuracy. Given substantial methodological concerns across studies, cautious interpretation is required, No firm clinical recommendation can be made regarding the use of standalone VLMs. Further research employing high-quality datasets is needed to ensure the reliability and clinical applicability of future VLMs.

## 1. Introduction

Artificial intelligence (AI) has advanced exponentially over the past decade, with progress accelerating markedly in recent years. Within the healthcare sector, AI integration has transformed medical practice and clinical research across domains ranging from microscopic pathology diagnosis to patient-care augmentation and hospital data management. A diverse array of architectures has been introduced, including traditional prediction models, large language models (LLMs), vision-language models (VLMs), and neural networks [1,2,3,4]. Among these, VLMs represent some of the most advanced tools by incorporating image data as a primary training source. This image data includes a variety of visual information related to patient condition, such as physical image from lesions, or image sections from radiologic procedures. Given the pivotal role of medical imaging in clinical diagnostics, the integration of VLMs into healthcare delivery offers substantial transformative potential.

While earlier applications of VLMs, such as report generation or basic image classification, prioritized image data [3,4,5,6,7], recent diagnostic models were designed to incorporate both textual and image inputs more synergistically [8,9,10,11]. The textual data in this context of novel VLMs is not merely image labels, but encompasses diverse patient structured and unstructured data, including patient history in clinical notes, laboratory results, reports, diagnoses, and interventions. Therefore, these multimodal VLMs were developed to analyze data more closely to clinicians, combining information from different modalities to deliver a diagnosis. Some of these emerging multimodal models have demonstrated promising diagnostic performance comparable to that of physicians [8]. However, some studies reported certain models underperformed relative to human benchmarks [10]. This discrepancy likely stems from heterogeneities in training datasets and specific architectural strategies for diagnostic task learning. Despite rapid progress of medical VLM research, the majority of prior systematic reviews of VLM focus on unimodal models using image data [12,13]. There remains a lack of systematic assessment of the performance and limitations of diagnostic VLMs trained on both patient image and textual data. A comprehensive evaluation of these models is essential to identify effective model architecture frameworks, current limitations, and key areas for further improvement.

In this study, we systematically evaluate the diagnostic performance of multimodal VLMs that integrate both clinical context and imaging data within the medical diagnostic process in hospital settings. Primary outcomes include diagnostic performance of multimodal VLMs compared to comparators, including other multimodal models, unimodal models, and clinicians. Secondary outcomes include diagnostic errors and time to diagnosis.

## 2. Materials and Methods

### 2.1. Search Strategy

This systematic review protocol was registered with the International Prospective Register of Systematic Reviews (PROSPERO; registration number: CRD420251244054). The review adhered to the Preferred Reporting Items for Systematic Reviews and Meta-Analyses (PRISMA) guidelines, CHecklist for critical Appraisal and data extraction for systematic Reviews of prediction Modelling Studies (CHARMS), and the Cochrane Collaboration Handbook [14,15,16]. The PRISMA 2020 checklist and the PICOTS (Population, Index model, Comparator model, Outcome, Timing, Setting, and intended use of the prediction model) criteria as recommended by the guidance of the Cochrane Prognosis Methods group are provided in Supplementary Data S1 [16,17].

A medical librarian (M.M.) developed and executed literature searches in consultation with the investigators. We used MeSH thesaurus terms from MEDLINE, EMTREE thesaurus terms from Embase, and free text words for search concepts related to visual language models and manually translated searches across resources using syntax, controlled vocabulary, and search fields. We originally deployed searches on 27 January 2025 and re-deployed them without changes on 17 December 2025.

We identified studies from searches of the databases Embase, MEDLINE, and Cochrane Central Register of Controlled Trials (CENTRAL)—all via the Wolters Kluwer Ovid interface—Scopus, and Science Citation Index Expanded (SCI-Expanded), Emerging Sources Citation Index (ESCI), and Preprint Citation Index (PCI)—all via the Clarivate Analytics Web of Science interface—and IEEE Xplore via IEEE. We applied English language and publication date limits. We applied subject areas in Scopus. We did not apply published search filters. We deduplicated records using a method by Bramer et al. [18]. We performed forward and backward citation searching of the bibliographies of included studies. We report search methodology according to PRISMA-S, an extension to the PRISMA statement for reporting literature searches in systematic reviews [19]. Detailed searches are provided in Supplementary Data S2.

### 2.2. Eligibility Criteria

We included studies that met all the following criteria: (1) used real-world data from adult patients (≥18 years); (2) evaluated multimodal VLMs integrating both patient textual data (e.g., patient history, physical examination, laboratory values, diagnosis, or treatments) and image data (e.g., computed tomography (CT), magnetic resonance imaging (MRI), X-Ray images, ultrasound images, or patient photo or video) for diagnostic purposes; (3) included comparators (human clinicians, other multimodal or unimodal models) or no comparator; (4) reported diagnostic-related outcomes (e.g., diagnostic accuracy, disease detection, diagnostic errors, time to diagnosis, or related performance metrics); and (5) were published after 2000 (when the development of modern machine learning frameworks was widespread). We excluded studies evaluating single-modality models (text or image only) as an intervention, those focusing solely on visual question answering (VQA) without real patient data, report generation, or pathology and histopathology images, case reports, case series, single abstracts and conference proceedings, editorials, commentaries, review articles, and non-English articles.

### 2.3. Study Selection

Based on the eligibility criteria, titles and abstracts were independently screened by three reviewers (L.E., L.O.G., and Y.S.). Full-text articles were then independently assessed by three reviewers for final eligibility. Discrepancies were resolved through discussion among all authors until consensus was reached.

### 2.4. Data Extraction and Quality Assessment

Data extraction for each study was independently performed by two of three reviewers (L.E., L.O.G., and Y.S.) using a standardized data collection form in accordance with the CHARMS checklist [16]. Disagreements were resolved through group discussion. The following variables were extracted: study title, names of authors, year of publication, study design, country of study, population, intervention information (including training models and training data), comparator, outcomes (including diagnostic performance, disease classification, disease detection, diagnostic errors, time to diagnosis), and data availability discussed in the study.

The methodological quality of each study was assessed independently by at least two investigators (L.E., L.O., and Y.S.). The Prediction model study Risk Of Bias Assessment Tool + AI (PROBAST + AI) was used to assess the quality and risk of bias of each included study [17,20,21]. Disagreement between investigators was settled by group discussions.

### 2.5. Data Synthesis and Analysis

When possible, we extracted or calculated accuracy, F1, and AUC as diagnostic outcomes. We planned to perform a meta-analysis of outcome data. However, given the large heterogeneity of study designs, populations, and interventions in the included studies, the pooling of effect estimates was not justified and hence abandoned. Instead, we narratively synthesized the data from the included studies using SWiM guidelines [22]. The eligible studies were grouped by the parameters which were considered the most likely sources of heterogeneity—comparators and model building methods.

## 3. Results

### 3.1. Study Characteristics (Table 1)

**Figure 1:**
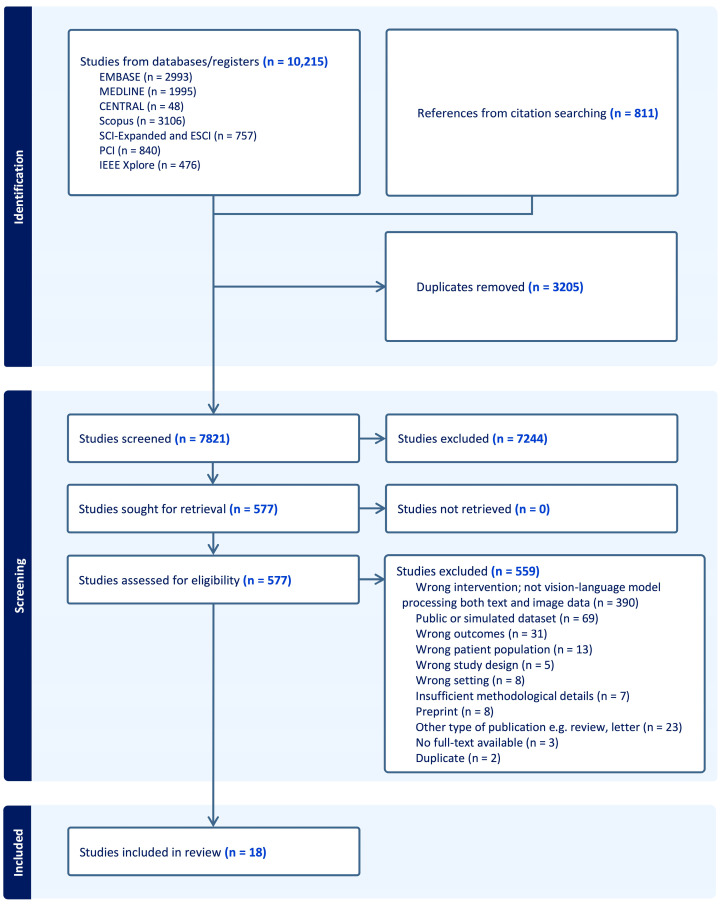
PRISMA flow of search methodology and selection process; CENTRAL, Cochrane Central Register of Controlled Trials; ESCI, Emerging Sources Citation Index; PCI, Preprint Citation Index; SCI, Science Citation Index.

A total of 11,026 studies were identified through the systematic literature search and additional searches, including gray literature search and reference mining [23]. After removing 3205 duplicate records, 7821 unique articles remained for title and abstract screening. Of these, 7244 articles were excluded due to publication type (e.g., reviews, or letters), or lack of relevance to the research topic. A total of 577 articles underwent full-text review, resulting in the exclusion of 559 records. Ultimately, 18 studies met the inclusion criteria and were included in the systematic review [8,9,10,11,24,25,26,27,28,29,30,31,32,33,34,35,36,37]. The study selection process and reasons for exclusion are presented in the PRISMA diagram (Figure 1).

**Table 1:** Characteristics of the included studies.

Study	Country, Study Design	Purpose of the Study	Population	Datasets	Model Name and Development Method If Available	Code and Data Availability
Studies comparing performance of VLMs and physicians						
Karaman et al., 2026 [10]	Turkey, single center, retrospective	To assess the diagnostic accuracy of 3 LLMs for schwannoma and meningioma	Patients with vestibular schwannoma or meningioma located in the CPA	53 patients Text: clinical data Image: 53 pairs of selected slices from CT and MRI	- ChatGPT-4o - Grok-3 - Claude 3.7 Sonnet Model building method not reported; proprietary system with closed-source development and training protocols.	https://github.com/peterhan91/Multimodal-Probes accessed on 2 March 2026
Sorin et al., 2025 [8]	Israel, single center, retrospective	To evaluate the performance of GPT-4V in an integrated analysis of ocular images and clinical text	Patients with ophthalmologic conditions	40 patients selected by physicians Text: selected patient information, e.g., clinical contexts Image: patient images	ChatGPT-4V; model building method not reported; proprietary system with closed-source development and training protocols.	Available from the corresponding author upon request
Tanyeri et al., 2025 [24]	Turkey, single center, retrospective	To evaluate the diagnostic performance of GPT-4o in emergency abdominal CT cases compared to radiology residents	Patients in emergency department	45 patients Text: summarized clinical description Image: CT	ChatGPT-4o; model building method not reported; proprietary system with closed-source development and training protocols.	Not publicly available but available from the author upon request
Wu et al., 2025 [25]	China, multicenter, RCT	To develop a model as a copilot for ophthalmology	Patients with eye diseases	Pretraining: - 14.5 million images in 5 modalities: CFP, OCT, UWF, FFA, and external eye photo - 0.4 million clinical texts Test-RCT in Experiment 3: 668 patients Text: clinical text Image: CFP, OCT, UWF, FFA, and external eye photo	EyeFM; the model was pretrained independently on an image module and then image-report pairs underwent joint vision-language pretraining with architecture of LLaVA. Subsequently, the model was trained by generated instruction-following data to handle conversational tasks. The model was further adapted to downstream tasks, including image downstream tasks and human-in-the-loop.	https://github.com/eyefm/EyeFM accessed on 2 March 2026
Studies comparing performance between different VLMs						
Legrain et al., 2025 * [26]	Belgium, single center, prospective	To investigate the performance of 3 LLMs in analyzing clinical pictures of common phoniatric disorders	Patients consulting for primary laryngeal symptoms	50 patients Text: clinical data Image: laryngostroboscopic images	- ChatGPT-4o - DeepSeek - Claude-3.7-Sonnet Model building method not reported; proprietary system with closed-source development and training protocols.	Not mentioned
Oikonomou et al., 2026 [11]	United States, multicenter, retrospective	To develop multimodal models combining EHR and ECG data for screening in health systems	Training: patients who underwent ECG and TTE Validation: patients who underwent ECG and CMR	Training: 159,322 patients Internal validation: 8979 External validation: 38,749 from 2 external datasets Text: EHR data Image: ECG and TTE	TARGET-AI; the model development utilized a two-stage multimodal approach: first, a longitudinal EHR foundation model (CLMBR-T) analyzed patient medical histories to identify high-risk candidates; second, a vision-language model based on ViT and contrastive learning interpreted ECG images to detect specific cardiac pathologies. This gatekeeping strategy was designed to ensure AI is only triggered for patients where it provides high diagnostic value.	https://github.com/CarDS-Yale/target-ai-shared accessed on 2 March 2026
Schramm et al., 2025 [27]	Germany, single center, retrospective	To evaluate the impact of varying multimodal inputs on the accuracy of GPT-4V-based brain MRI differential diagnosis	Patients with brain MRI images	60 patients selected by physicians Text: brief medical history, image description Image: 60 MRI	ChatGPT-4V; model building method not reported; proprietary system with closed-source development and training protocols.	Not mentioned
Sun et al., 2026 [9]	China, single center, retrospective	To evaluate the diagnostic performance of GPT-4o in brain tumor diagnosis	Pre-operative brain tumor patients	239 patients Text: brief clinical history Image: MRI slice	ChatGPT-4o; model building method not reported; proprietary system with closed-source development and training protocols.	Available from the corresponding author upon request
Wang X et al., 2025 [28]	Singapore, single center, retrospective	To develop a multi-modality attention network integrating MPI and clinical data for CAD evaluation	Patients who underwent MPI scans	1468 patients Text: clinical variables Image: 1072 MPI	The developed network uses ICCA to fuse stress/rest MPI images and CDGA clinical data via attention modules to integrate 39 clinical variables. Models were pretrained with MAE. A masked autoencoder was employed as the foundation to enrich the feature representation within MPI.	Not publicly available
Xu et al., 2025 [29]	China, multicenter, cross-sectional	To develop an automated multimodal AI model (text/audio/video) for depression detection	MDD patients and healthy volunteers	Training and validation: 289 patients External validation: 100 patients Text, audio, and video data are from interview videos	Textual data: Multi-round interview transcripts (MINI interview; chatbot symptom interview; brief affective interview). Text encoder: XLNet. Conversation tokens joined into continuous sequences. Audio data: Interview audio files were segmented by sliding window into segments; audio encoder: Chinese-HuBERT; segment representations averaged over time. Visual data: Interview videos were segmented into clips; visual encoder: VideoMAE with LGI-Former. Fusion/model: Multi-head cross-attention mechanism to integrate text, audio, and video; final subject-level prediction obtained by voting across multiple instances per subject. Chatbot dialog topics derived from HAMD-24 and HAM-A; GPT-based dialog system fine-tuned using >300 clinician-rated HAMD interview audio files transcribed to text.	Data and code availability not explicitly provided
Studies comparing performance between multimodal models and unimodal models						
Hooshangnejad et al., 2024 [30]	United States, single center, retrospective	To develop novel EHR-guided tumor detection via auto-segmentation method	Training and validation: LIDC-IDRI public data with 201 CT Test: Patients with lung tumor	Tested on 10 patient EHR data from hospital database Text: EHR data Image: MRI	EXACT-Net; a multimodal framework combined a 3D UNet-based CT segmentation model with a zero-shot LLM that extracted tumor location (lung lobe) from EHRs. The segmentation model first detected all possible tumor regions, and the LLM-provided lobe information was then used to filter out false positives by masking predictions outside the reported lobe.	Upon request from authors
Li X et al., 2025 [31]	China, multicenter, cross-sectional	To develop a DL-based visual and multimodal detection framework for CAD using retinal images	Patients who underwent successful CAG	383 patients Text: 45 clinical indicators Image: OCT/OCTA	The model extracts multimodal features from retinal images and clinical data. Three identical ResNet34 networks process OCT, OCTA, and Projection Map images. Clinical information is encoded in two ways: numerical data are processed by an MLP to generate a feature vector, and the same numerical data are converted into clinically meaningful text and encoded using BiomedBERT. A cross-modal attention module enables information exchange across modalities, then fused features are aggregated and fed into a classifier for final prediction.	Not mentioned
Ma et al., 2025 [32]	China, multicenter, cross-sectional	To develop models for diagnosing ophthalmic diseases	Patients with ophthalmic diseases	Development: 90 cases Internal validation: 2292 External validation: 2940 Text: real dialogs with clinical data Image: slit-lamp and smartphone-acquired images	IOMIDS; a dynamic prompt system was designed to enhance ChatGPT’s role in patient consultations by integrating both textual and image data. - Text Model: trained using real dialogs categorized by chief complaints; 90 cases selected for prompt construction. - Image Models: trained on respective image sets. Multimodal Integration: - Defined thresholds to exclude irrelevant diagnoses from image outputs; combined text prediction with imaging results via diagnosis prompts; for combined modality (both images), models used a union rule to improve accuracy.	Not publicly available
Schmidl et al., 2025 [33]	Germany, single center, cross-sectional	To assess LLM’s ability to differentiate between SCC, premalignant lesions, and benign or lesion-free conditions	Patients with or without oral lesion	45 patients Text: clinical data Image: 45 lesion images	ChatGPT; model building method not reported; proprietary system with closed-source development and training protocols.	As available in the publication
Wang Y et al., 2025 [34]	China, multicenter, prospective	To develop a DL model based on DMUS video for the differential diagnosis of benign and malignant SPNs	Participants with subpleural pulmonary nodules	372 participants Training: 154 Validation: 39 Internal Test: 88 External Test: 91 Text: clinical information Image: US video	The model integrated DMUS videos (Grayscale Ultrasound and Contrast-Enhanced Ultrasound) and clinical information including sex, age, smoking history, and respiratory diseases. It integrated perfusion intensity variations and spatiotemporal features, and had 2 innovative branches: TICA and DMVU.	https://github.com/JoSeunghyr/TIC-LUV accessed on 2 March 2026
Zeljkovic et al., 2025 [35]	Croatia, single center, cross-sectional	To evaluate GPT-4’s ability to interpret 12-lead ECGs with and without clinical context	Adults in hospital setting from CaRD registry; 150 12-lead ECGs	150 cases Text: clinical scenario Image: 150 ECG images	ChatGPT-4; model building method not reported; proprietary system with closed-source development and training protocols.	De-identified data from CaRD registry
Studies conducted without comparator						
Chiesa-Estomba et al., 2025 * [36]	Spain, single center, retrospective	To evaluate ChatGPT-4o in analyzing clinical fiberoptic videos of suspected laryngeal malignancies compared to expert clinicians	Patients consulting for primary laryngeal disease	20 patients Text: clinical history Image: CT and laryngeal fiberoptic video examinations	ChatGPT-4o; model building method not reported; proprietary system with closed-source development and training protocols.	Not mentioned
Li J et al., 2023 [37]	China, single center, retrospective	To develop an interpretable AI framework for diagnosing pulmonary diseases using multimodal data	Patients with lung disease	EHR data from 1000 patients Text: 1000 EHR data from hospital database Image: 1000 CXR	The model integrates clinical text from EHRs and CXR imaging features extracted via a VLM into a hierarchical patient graph, which is then processed by a Graph Neural Network for pulmonary disease classification. Prototype learning provides intrinsic interpretability by comparing patients to representative disease examples, while counterfactual reasoning was used to provide key features influencing each diagnosis.	Not mentioned

* Article in press; AI, artificial intelligence; BERT, Bidirectional Encoder Representations from Transformers; CAD, coronary artery disease; CAG, coronary angiography; CaRD, Cardiology Research Dubrava registry; CDGA, clinical data-guided attention; CFP, color fundus photography; CLIP, Contrastive Language–Image Pre-training; CLMBR-T, Clinical Language Modeling-based Representations using Transformers; CMR, cardiac magnetic resonance imaging; CPA, cerebellopontine angle; CT, computed tomography; CXR, chest X-Rays; DL, deep learning; DMUS, dual-modality ultrasound; DMVU, Dual-Modality Video Understanding; ECG, electrocardiogram; EHR, electronic health record; HAM-A, Hamilton Anxiety Rating Scale; HAMD, Hamilton Depression Rating Scale; FFA, fundus fluorescein angiography; ICCA, image-correlated cross-attention; LGI-Former, local-global interaction Transformer; LLaVA, Large Language and Vision Assistant; LLM, large language model; LIDC-IDRI, Lung Image Database Consortium imaging collection; MAE, masked autoencoder; MDD, major depressive disorder; MINI, Mini International Neuropsychiatric Interview; MLP, multilayer perceptron; MPI, myocardial perfusion imaging; MRI, magnetic resonance imaging; OCT, optical coherence tomography; OCTA, optical coherence tomography angiography; RCT, randomized controlled trial; SCC, squamous cell carcinoma; SPN, subpleural pulmonary nodules; TICA, Intelligent Time-Intensity Curve Analysis; TTE, transthoracic echocardiogram; US, ultrasound; UWF, ultra-widefield imaging; ViT, Vision Transformer; VLM, vision-language model.

All 18 included studies reported the utilization of VLMs processing both text and image data and were either published or indicated as accepted for publication from 2023 to 2026 [8,9,10,11,24,25,26,27,28,29,30,31,32,33,34,35,36,37]. Nine studies performed both model development and evaluation [11,25,28,29,30,31,32,34,37], and the remaining nine studies only conducted model evaluation on previously established models [8,9,10,24,26,27,33,35,36]. Eleven studies were conducted in Asia [8,9,10,24,25,28,29,31,32,34,37], five in Europe [26,27,33,35,36], and two in North America [11,30]. The general characteristics of the included studies are presented in Table 1.

All included studies utilized real-world patient data obtained from electronic medical records (EMRs) or research-specific clinical datasets. For textual data, various types of clinical information were leveraged, including demographics, chief complaints, symptoms, and physical examination findings. Among these, only three studies explicitly reported that patient textual data were extracted directly from EHRs [11,30,37]. The included studies utilized a range of imaging modalities, including X-Rays, CT, MRI, ultrasound, and clinical photographs of physical lesions. These visual data comprised both static images and video sequences. A detailed summary of the included studies is presented in Table 1.

The included studies spanned diverse medical specialties, including psychiatry, pulmonology, emergency medicine, otolaryngology, ophthalmology, cardiovascular disease, and radiology. Notably, most of these studies did not report baseline patient characteristics, such as race, age, sex, or comorbidities. Regarding model performance, most studies reported the primary output as a single correct diagnosis [8,9,10,11,24,25,26,28,29,31,32,33,34,35,36,37]. The remaining two studies reported alternative metrics: one reported the nodule detection rate [30], while another focused on differential diagnosis accuracy [27] (Table 1).

All included studies disclosed no financial relationships or conflicts of interest with the corporations owning the commercial models evaluated in the studies.

### 3.2. Methodological Quality and Risk of Bias

For an assessment of overall quality concern of model development, five development studies were rated as high concern, three were rated as unclear concern, and the remaining study was rated as low concern.

For overall applicability concern of model development, three studies were rated as high concern, and six studies were rated as low concern.

Regarding overall risk of bias of model evaluation, thirteen studies were rated as high concern, two were rated as unclear level of concern, and the remaining three studies were rated as low concern. The observed high risk of bias was primarily due to high concern ratings regarding dataset quality, including small sample size, underreported confounding control, and a lack of external validation. A detailed evaluation using the PROBAST + AI tool is presented in Supplementary Data S3.

For overall applicability concern of model evaluation, five studies were rated as high concern, five were rated as unclear level of concern, and the remaining eight studies were rated as low concern.

The results of the overall evaluation for each study are presented in Table 2.

### 3.3. Performance Outcomes of Reported Multimodal VLMs

We categorized the included studies with respect to comparators to elucidate performance of VLMs compared to human clinicians and other models. The summarized information of the outcomes of the included studies is presented in Table 3.

#### 3.3.1. Performance of Multimodal VLMs Related to Physicians

Across 18 studies, four compared diagnostic performance between VLMs and human physicians [8,10,24,25].

Three out of four studies demonstrated a direct comparison of performance of VLMs with that of physicians [8,10,24]. All three studies evaluated established models, including ChatGPT, Grok, and Claude, with ChatGPT models being featured in all three studies. Two studies, one involving ophthalmic diseases and another conducted in an emergency department setting, reported comparable diagnostic performance between VLMs and physicians, with slightly higher accuracy from human physicians [8,24]. Notably, one of them also reported that ChatGPT-4V with clinical context achieved higher performance compared with an identical model without clinical context (67.5% vs. 47.5%, *p* = 0.033) [8]. In contrast to these two studies, another study demonstrated lower accuracy from three VLMs (18.9–52.8%) compared to a radiologist and neuroradiologist (79.2% and 92.5% respectively) [10].

The fourth study developed the EyeFM model to be a copilot in ophthalmic disease diagnosis and assessed the performance of physicians with and without VLM assistance [25]. This study reported higher accuracy in a group of physicians with EyeFM deployment compared to physician performance without model assistance (92.2% vs. 75.4%).

#### 3.3.2. Performance of Multimodal VLMs Compared to Other Models

##### Performance Between Different Multimodal VLMs

Six studies in various disease domains, including laryngology, cardiovascular diseases, neurology, radiology, and psychiatry, reported performance between VLMs [9,11,26,27,28,29]. Of these, five studies were conducted with newly developed models and demonstrated higher performance from full VLMs compared with models trained in similar environments but using fewer modalities [9,11,27,28,29]. These findings are further addressed in the Section 4.

Only one study focusing on laryngeal diseases was conducted with publicly available models with presumably different model architecture [26]. These models included ChatGPT-4o, DeepSeek, and Claude-3.7. In this study, all models demonstrated low performance with diagnostic accuracy lower than 30% [26].

##### Performance Between Multimodal VLMs and Unimodal Models

In this category, six studies comparing multimodal VLMs with unimodal models processing either text or image data demonstrated higher diagnostic accuracy from multimodal VLMs [30,31,32,33,34,35]. Of these, five studies developed VLMs for diagnostic classification [31,32,33,34,35] and the remaining study was conducted to improve image detection accuracy [30].

Li et al. developed a model utilizing EHR-derived clinical texts to enhance tumor nodule detection on imaging [30]. The study reported a 50% increase in diagnostic accuracy of the VLM compared to a model with an identical framework but without EHR data input (EXACT-Net with EHR data vs. EXACT-Net without EHR data: 70% vs. 20%) [30].

Two studies were conducted with VLMs without any comparison [36,37]. A study assessing the capability of ChatGPT-4o to diagnose malignant and premalignant laryngeal diseases reported an accuracy of 30% [36]. Another study in the population with pulmonary diseases developed a VLM with high diagnostic performance (F1 87.7% and accuracy 87.8%) [37].

### 3.4. Categories of Multimodal VLMs Respective to Source of Dataset

#### 3.4.1. VLMs Using Real Medical Record Data

Of all included studies, three studies featured VLMs that extracted text and image data from the EHRs [11,30,37]. All three studies included both model development and evaluation. Of these, one study used structured and unstructured data, including chief complaints, patient history, and diagnoses from clinical notes [37]. Another study extracted only unstructured data from clinical reports [30], and the remaining study mainly used coded medical events as the text dataset, including diagnosis, laboratory values, medications, and procedures [11]. The image data used in these studies included X-Rays, CT, MRI, electrocardiogram (ECG), and transthoracic echocardiogram (TTE).

The fact that only a minority of the included studies utilized data directly from EHRs implies that current VLM literature heavily relies on curated, pre-processed datasets. Consequently, these models may potentially underperform when deployed within real-world clinical databases and EHR workflows.

#### 3.4.2. VLMs Using Real Patient Information Outside Medical Records

There were 15 included studies conducted with patient data obtained outside of medical records [8,9,10,24,25,26,27,28,29,31,32,33,34,35,36]. Text datasets included selected clinical texts derived via research-format collection, patient dialogs with physicians, and videos. Of these, one study collected patient data through interviews recorded in video format which were subsequently transcribed into text, audio, and visual data [29].

### 3.5. Categories of Multimodal VLMs Respective to Medical Specialty

We classified the included studies into their respective specialties to examine characteristics within similar populations as detailed in Supplementary Data S4.

#### 3.5.1. Cardiovascular Diseases

Four studies focused on cardiovascular diseases [11,28,31,35]. Of these, two studies evaluated model performance in CAD diagnosis [28,31]. Although both targeted the same diagnostic endpoint, their populations, imaging modalities used, and model building methods were distinct. One study used retinal images from patients who underwent coronary angiography [31], while another utilized myocardial perfusion imaging (MPI) for development [28]. The two remaining studies incorporated ECG in architecture but were conducted in entirely different cohorts: one involved patients who underwent TTE [11] while another used retrospective data from a registry [35].

#### 3.5.2. Ophthalmology

Three studies evaluated models for ophthalmic disease diagnosis [8,25,32]. One study developed a copilot model [25] and two other studies evaluted standalone agents [8,32]. These studies with standalone agents used different architectures, images and clinical texts; one evaluated proprietarily built models incorporating real physician–patient dialogs, images of slit-lamp examination, and smartphone-acquired images [32] while another used commercial agents to process physician-made clinical descriptions and images of ocular lesions [8].

#### 3.5.3. Radiology

Three studies were conducted in the radiology department [9,10,27]. While all studies in this group are similar in using commercial models on brain tumor diagnosis, their populations, types of model, comparators, and outcomes of interest were different. For instance, two studies reported singular diagnosis outcomes but with distinct comparators, such as other models [9] or clinicians [10]. The remaining study uniquely reported differential diagnosis as an outcome [27].

#### 3.5.4. Otolaryngology

There are three studies focusing on otolaryngologic diseases [26,33,36]. All three featured ChatGPT as one of the models they evaluated. However, their image data were highly distinct, including laryngostroboscopic images [26], laryngeal fiberoptic videos [36], and images of oral physical lesions [33].

#### 3.5.5. Pulmonary Diseases

Regarding lung diseases, three studies developed models with highly distinct diagnostic tasks [30,34,37]. One study aimed at a detection task based on an auto-segmentation function [30], one evaluated binary classification between benign and malignant subpleural pulmonary nodules [34], and one assessed model performance in diagnosing four different lung diseases, including pneumonia, tuberculosis, lung cancer, and pulmonary embolism [37].

#### 3.5.6. Other Specialties

The two remaining studies were conducted in other settings. One study was conducted in the emergency department setting [24] and another in psychiatry [29].

## 4. Discussion

In this systematic review, we evaluated the diagnostic performance of multimodal VLMs using clinical context and imaging data across different clinical settings and comparator groups. Overall, multimodal VLMs consistently outperformed unimodal models using either text or image data alone. Performance of diagnostic VLMs as a standalone agent compared to humans remains inconclusive. Notably, one high-quality study demonstrated improved diagnostic accuracy when physicians used a VLM as an assistant. However, these findings should be interpreted with caution due to substantial methodological limitations and a high risk of bias across studies.

While recent advances in AI are poised to transform every sector of human operation including medical practice, multimodal information processing and clinical reasoning have long been fundamental components of physician expertise. These capabilities allow clinicians to synthesize complex data for effective clinical decision-making.

### 4.1. Multimodal VLMs and Clinical Reasoning

Early-generation VLMs relied primarily on a single training modality, image data, with textual inputs serving only as tokens or prompts to guide image-based training [3,5,6]. Similarly, earlier diagnostic VLMs developed for classification tasks utilized architectures that predominantly processed visual information [3,4]. Subsequently, novel VLMs were designed to integrate multimodal data from images and patient-specific textual information, such as clinical notes, diagnostic codes, and laboratory results retrieved from EHRs [25,38,39]. Across diverse medical settings and clinical problems, multiple studies demonstrated that multimodal models outperformed unimodal models via ablation studies or direct comparison [8,9,11,27,28,29,30,31,32,33,34,35]. These results demonstrated that models utilizing identical architecture achieved higher accuracy when granted access to both images and clinical text data. Conversely, restricting data modality resulted in lower model performance.

Beyond diagnostic classification, multimodal VLMs enhance specialized detection capabilities. For instance, in a study developing a tumor nodule detection model augmented by EHR data, performance improved significantly when contextual information (e.g., the specific side and lobe of the lung containing the lesion) was provided [30]. This mirrors the clinical practice where physicians focus their attention on specific anatomical regions based on prior clinical findings. Furthermore, recent research has introduced models capable of generating diagnostic rationales, reflecting the human process of inspection and reasoning [40].

Ultimately, these findings suggest that a multimodal approach overcomes the inherent limitations of single-modality models.

### 4.2. Implications for Diagnostic Workflow and Efficiency

Prior to the significant integration of AI in healthcare, automation served as a key driver of medical advancement across various domains, including guideline development, research, emergency protocols, and EHR technology. With the emergence of AI, including novel VLMs, automation is increasingly being incorporated directly into the diagnostic workflow.

In clinical practice, several modifiable “time lags” exist between the initiation of a diagnostic workup and the delivery of treatment. Even when medical history is obtained and imaging is available, a diagnosis is not instantaneous; it requires a physician to retrieve, synthesize, and interpret the data. This temporal window is influenced by personnel capacity, professional roles, and clinical workload.

Multimodal VLMs may offer the potential to optimize clinical workflows by assisting in high-throughput diagnostic tasks. Within the modern electronic health record (EHR) system, a high volume of longitudinal data, including patient history, medical imaging, laboratory results, and diagnoses, is collectively stored. The deployment of VLMs within these systems could facilitate continuous model refinement and enable the near-instantaneous delivery of diagnostic outputs. For instance, precisely developed models could generate differential diagnoses or preliminary findings immediately upon the availability of imaging and clinical notes in the EHR. Furthermore, these applications may mitigate human errors stemming from cognitive bias and physical fatigue. Consequently, these advantages can support care teams operating in high-acuity settings characterized by high patient risk and massive data volumes, such as the intensive care unit (ICU) [41,42,43]. However, it is critical to acknowledge that AI tools can introduce their own risks to the workflow including automation bias, affirmation bias, and potential care disparities [44,45,46].

For an assessment of automation capability, we sought to examine studies that quantified the “time to diagnosis” achieved by these models relative to standard human performance. Notably, no studies included in this review reported this specific outcome. Future research evaluating this metric is essential for assessing the true impact of VLM-driven automation on medical diagnoses and patient outcomes.

### 4.3. Comparison with Clinician Performance and Role of Diagnostic VLMs in Clinical Practice

It is essential to evaluate the efficacy of VLMs relative to human clinicians. Recent research featuring multimodal VLMs has reported conflicting results regarding their performance compared to that of physicians [8,10,24]. Two studies reported comparable diagnostic accuracy between ChatGPT models and physicians [8,24], while another study demonstrated lower performance across three established models when compared to radiology residents and neuroradiologists [10]. These studies were assessed to have high concerns for risk of bias due to non-transparent training methodologies and a lack of accessible information on training data of these commercially available models. Given these limitations, results should be interpreted with caution.

Interestingly, one randomized controlled trial evaluated a VLM as a clinical assistant by comparing physician diagnostic performance with and without VLM support [25]. In this trial, ophthalmologists in the intervention arm received VLM-generated diagnostic suggestions prior to finalizing their assessments while the control group operated without VLM assistance. The study found that physicians achieved higher diagnostic performance when augmented by the VLM [25]. This finding underscores the potential of VLMs to serve as clinical decision support systems.

Synthesizing these observations, while evidence remains conflicting regarding the reliability of VLMs as standalone diagnostic agents, their role as clinician “copilots” appears to enhance diagnostic precision. It is important to note that this specific copilot was specifically built for medical tasks [25], whereas other studies utilized general-purpose commercial models with proprietary architectures and training datasets non-specific to the medical domain [8,10,24]. Considering this, model architecture and training data could be critical determinants of model accuracy and clinical utility.

### 4.4. Architecture and Development Strategies

#### 4.4.1. Model Development

**Figure 2:**
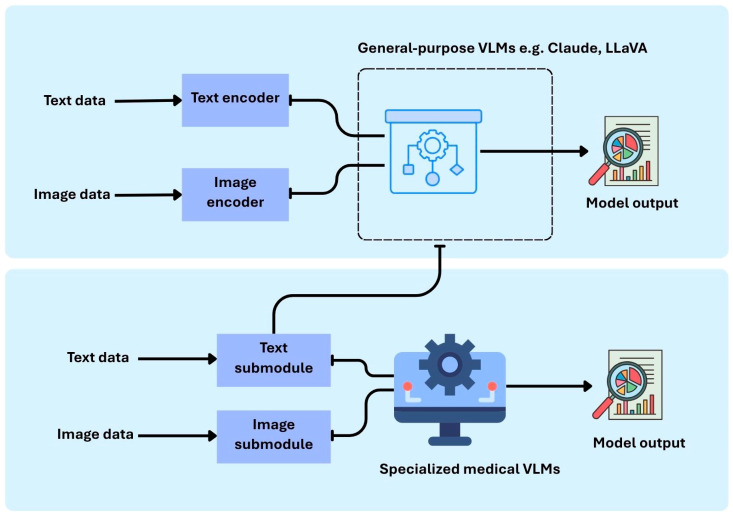
Novel architecture of multimodal diagnostic VLMs with submodule models; VLMs, vision-language models.

Advancements in model building methods now enable complex integration of multimodal datasets [7,47]. These innovations provide a robust framework for task-specific diagnostic tools by incorporating text and image processing modules together into a singular system. Frequently utilized frameworks include LLM, Large Language and Vision Assistant (LLaVA), Bidirectional Encoder Representations from Transformers (BERT), and Vision Transformer (ViT). While general-purpose models, e.g., ChatGPT, Gemini, Claude, or open-source LLaVA, are often utilized as standalone agents, specialized proprietary architectures have begun incorporating these models as submodules (Figure 2). This approach enables integration with specialized medical encoders, resulting in superior multimodal performance [30,32]. Another established strategy for adapting generalist models to medical tasks involves domain-specific pretraining on medical datasets [25,32]. This method allows the architecture to preserve foundational abilities, such as image boundary detection or natural language syntax, while learning specialized diagnostic patterns from medical data.

Beyond module selection, the specific training sequence and layer architecture significantly influence model efficacy. For instance, a study in cardiovascular diseases evaluated VLMs using different frameworks for incorporating EHR textual data [25]. Results indicated that the targeted strategy (risk stratification by textual EHR variables before imaging integration) consistently outperformed the untargeted approach (combining text and image data without preliminary risk stratification) [25]. Another study developed multimodal models combining textual data, patient speech audio, and video recordings of facial expressions to assess depression risk [48]. While emotion signs alone are insufficient for a clinical diagnosis of depression, this study demonstrates that VLMs can be engineered to classify complex, subjective features such as affect.

Collectively, these findings suggest that model architecture greatly influences diagnostic performance. However, as each methods possess unique strengths and structural constraints, a deliberate selection of methods or modules is required to tailor the best configuration to the specific clinical task.

#### 4.4.2. Dataset Quality

Regardless of model complexity, data sources remain the fundamental determinant of model performance. High-quality datasets are essential for developing accurate and robust models. Most studies utilized public datasets for model training [38,40,49,50,51,52,53,54,55,56,57,58,59]. While the use of public repositories is common in AI research due to their accessibility, it can introduce methodological risks. Similar to traditional clinical research, the use of retrospective, pre-existing data can introduce biases related to missing values and uncertain data reliability, which limits the ability to verify data quality and model outcomes.

Our study restricts inclusion criteria to only include studies with real-world datasets and exclude those with models solely trained on public datasets, which typically lack the component of real-world heterogeneity. We conducted our investigation in this manner to minimize data-driven biases and to identify studies that possess an intrinsic advantage in dataset composition, thereby demonstrating a higher probability of translation to clinical deployment. However, it is important to acknowledge that real-world datasets are also inherently prone to biases. Consequently, rigorous methodology and transparent quality assessments are paramount to ensuring the clinical validity and high quality of the evaluated models.

Through the PROBAST + AI assessment, the majority of the included studies were rated as high concern for dataset quality despite using real-world datasets (Table 3). The participant section emerged as the most common source of bias (Supplementary Data S3). Small sample size, potential confounding, and a lack of external validation were among the most prevalent determinants accounting for the high-concern ratings. Only one study adequately addressed confounding [25]. These findings underscore the need for more rigorous population selection and transparent reporting of study protocols in the field of medical informatics. In addition, the quality assessment tool plays a crucial role in determining the study reliability. Expanding the current tool to include targeted criteria may be required to address specific risks of bias unique to real-world clinical studies.

### 4.5. Current Methodological Quality Assessment of AI Model Study

To thoroughly assess a diagnostic model study, numerous guidelines and assessment tools, e.g., PROBAST + AI, have been developed to address areas prone to bias and error, including dataset and predictor quality, model development methods, the handling of missing data, and overfitting risk [16,17,60,61]. However, as these guidelines were primarily designed for prediction model studies, they often lack specific recommendations for evaluating risks of bias inherent to clinical research, such as confounding factors, sample randomization, or population heterogeneity. Therefore, the assessment of AI-based prediction models should also integrate frameworks commonly applied in traditional clinical research into additional sections of assessment to address these risks of bias. We propose that this adjustment may be applied to the subsection of quality and risk of bias of study participants, or added as a new dedicated assessment related to clinical studies.

### 4.6. Safety, Security and Implementation Considerations

Given the observed developments and novel findings, the implementation of VLMs in medical practice continues to expand. Future iterations may be developed for real-time deployment within electronic medical record systems. Recent studies have deployed and validated LLMs and other machine learning (ML)-based models in the EHR system [62,63]. Consequently, addressing safety and security concerns regarding the use of these models is paramount. While the U.S. Food and Drug Administration (FDA) has published regulatory frameworks for artificial intelligence-powered healthcare products, including model development recommendations, machine learning-based device action plans, and established criteria for AI-enabled medical device approval, critical limitations persist. Specifically, vulnerabilities remain regarding real-time monitoring, extensive post-market evaluations of commercial software, and the transparent disclosure of training data source [64]. Consequently, it is essential for model developers and healthcare institutions to collaborate in executing rigorous quality assurance protocols and adhering to mandatory regulations to safeguard patient privacy and clinical safety.

Among the studies included in this review, none addressed these topics. However, as these tools have the potential for integration into environments containing sensitive patient data, such as local hospital systems or interhospital networks, the risk of data compromise cannot be ignored. System security must be ensured. Another critical safety consideration is the assessment of model performance over time, particularly for models continuously deployed in active clinical environments. Regular maintenance and assessment of potential biases emerging from model usage are crucial for ensuring long-term efficacy and the safety of patient care.

### 4.7. Strengths and Limitations

This study possesses several notable strengths. First, it uniquely focuses on multimodal VLMs that integrate clinical context with imaging data across diverse medical settings. These multimodal models have not been systematically evaluated previously. By aggregating evidence from diverse clinical settings and specialties, our findings offer enhanced generalizability and provide a broader perspective on the utility of VLMs within the diagnostic process. This broad scope further facilitates the identification of common limitations identified across studies regardless of settings, including small sample size, a lack of external validation, and the need for clinical deployment studies. Second, we included only studies utilizing real-world patient data. This approach allows for a more rigorous evaluation of models while mitigating the inherent biases often observed in the majority of studies that leverage public datasets, including missing data, and limited real-world validity. Finally, by examining VLM performance across different comparator groups, including clinicians, multimodal models, and unimodal models, this review provides a nuanced understanding of the current capabilities of these models within the diagnostic process.

Our study also has several limitations that must be considered. First, while this review covers a wide range of diseases and settings, high heterogeneity across the included studies limited our ability to conduct a formal statistical meta-analysis. Further investigation with more restricted eligibility criteria, e.g., specific disease areas, may provide more focused insights. Second, the exclusion of non-English articles introduces a potential geographic and language bias. However, this restriction was to ensure consistency in evaluating VLM performance, as the majority of state-of-the-art diagnostic VLMs evaluated in the current literature utilize text encoders or tokenizers trained predominantly on English databases. Future research evaluating how cross-lingual models adapt to multi-language clinical environments will be essential to provide deeper nuance and broader validation in this domain. Third, most studies were found to have high concerns regarding methodological quality and a risk of bias. In addition, the performance metrics reported across the included literature were highly inconsistent, with several studies failing to report *p*-value. Taken together, a reliable interpretation of their results is limited. Future studies with high-quality study designs and robust, high-quality datasets are urgently needed. Lastly, research in this field is rapidly evolving. To remain current with emerging findings, a living systematic review would offer substantial benefit.

## 5. Conclusions

This systematic review demonstrates that novel diagnostic VLMs trained on multimodal datasets possess the potential to augment patient care by automating data-driven diagnosis. Multimodal models processing both text and image data consistently outperformed unimodal models. However, model performance compared to that of clinicians remains inconclusive. Current evidence suggests that VLMs perform a beneficial role as assistants to human physicians, and the customization of model frameworks with multimodal data could further enhance their efficacy. Notably, none of the included studies explicitly addressed patient safety or data security considerations. The development of standardized frameworks that rigorously mandate these protocols is essential. While the included studies highlight multiple directions for future development, the current evidence must be interpreted with caution, and no firm clinical recommendations regarding the standalone deployment of these models can be made due to substantial methodological concerns. Further research employing high-quality datasets alongside rigorous study designs to control the risk of bias is warranted to ensure the long-term reliability and clinical applicability of future VLMs.

## Figures and Tables

**Table 2:** Quality assessment of included studies with PROBAST + AI tool.

Study	Overall Judgment of Model Development		Overall Judgment of Model Evaluation	
	Quality Concern	Applicability Concern	Risk of Bias	Applicability Concern
Karaman et al., 2026 [10]	N/A	N/A	High	High
Sorin et al., 2025 [8]	N/A	N/A	High	Unsure
Tanyeri et al., 2025 [24]	N/A	N/A	High	Unsure
Wu et al., 2025 [25]	Low	Low	Low	Low
Legrain et al., 2025 [26]	N/A	N/A	High	Low
Oikonomou et al., 2026 [11]	Unsure	Low	Unsure	Low
Schramm et al., 2025 [27]	N/A	N/A	High	High
Sun et al., 2026 [9]	N/A	N/A	High	Unsure
Wang X et al., 2025 [28]	High	Low	High	Low
Xu et al., 2025 [29]	Unsure	Low	Unsure	Low
Hooshangnejad et al., 2024 [30]	High	High	High	High
Li X et al., 2025 [31]	High	High	Low	High
Ma et al., 2025 [32]	High	Low	High	Low
Schmidl et al., 2025 [33]	N/A	N/A	High	Unsure
Wang Y et al., 2025 [34]	Unsure	Low	Low	Low
Zeljkovic et al., 2025 [35]	N/A	N/A	High	Low
Chiesa-Estomba et al., 2025 [36]	N/A	N/A	High	Unsure
Li J et al., 2023 [37]	High	High	High	High

N/A: Not applicable.

**Table 3:** Outcomes of the included studies.

Study	Model output	Interventions	Comparators	Results	Notes
Studies comparing performance of VLMs and physicians					
Karaman et al., 2026 [10]	Diagnosis: vestibular schwannoma vs. meningioma	ChatGPT-4o Grok-3 Claude 3.7 Sonnet	Resident radiologist Experienced neuroradiologist	F1 64.8%, Accuracy 52.8% F1 29.5%, Accuracy 18.9% F1 55.1%, Accuracy 41.5% F1 83.3%, Accuracy 79.2% F1 **92.5%**, Accuracy **92.5%**	*p*-value not reported Models with clinical + expert textual imaging feature input achieved consistently higher accuracy compared to models with clinical + raw image input
Sorin et al., 2025 [8]	Diagnosis of ophthalmic diseases	GPT-4V with clinical context	GPT-4V without clinical context Non-ophthalmologic physician 1 Non-ophthalmologic physician 2	Accuracy 67.5% * Accuracy 47.5% Accuracy **72.5%** Accuracy 67.5%	* *p* = 0.033 compared with ChatGPT-4V without clinical context, *p* = 0.688 compared with physicians
Tanyeri et al., 2025 [24]	Diagnosis in emergency department setting	ChatGPT-4o	Advanced-experienced resident group Intermediate-experienced resident group Limited-experienced resident group	Accuracy 82.22% Accuracy 83.33%, *p* = 1.000 Accuracy **85.56%,** *p* = 0.549 Accuracy 75.56%, *p* = 0.605	*p*-value from comparisons of ChatGPT and resident groups
Wu et al., 2025 [25]	Diagnosis of ophthalmic diseases	EyeFM + physicians	Physicians alone	Accuracy **92.2%** Accuracy 75.4%, *p* < 0.001	
Studies comparing performance between different VLMs					
Legrain et al., 2025 * [26]	Diagnosis of laryngeal diseases	ChatGPT-4o	DeepSeek Claude-3.7	Accuracy 25.6% Accuracy 15.4% Accuracy **28.2%**	*p*-value not reported
Oikonomou et al., 2026 [11]	Diagnosis of patients with ECG	Targeted strategy (EHRs then AI-ECG)	Untargeted strategy (AI-ECG + EHRs) Untargeted strategy (AI-ECG alone)	F1 **0.54–0.89** F1 0.12–0.57 F1 0.09–0.58	*p*-value not reported External validation also showed highest accuracy from targeted strategy
Schramm et al., 2025 [27]	Differential diagnosis of brain MRI in neuroradiology	I + A + H + D	I + D I + H I + A I H + D D	Accuracy **69%** Accuracy 59%, *p* = 0.62 Accuracy 28%, *p* < 0.001 Accuracy 1%, *p* < 0.001 Accuracy 2%, *p* < 0.001 Accuracy 65%, *p* = 0.99 Accuracy 66%, *p* > 0.99	I = image A = annotation H = medical history D = image description *p*-value calculated from comparisons of (I + A + H + D) and other models
Sun et al., 2026 [9]	Brain tumor diagnosis: Final diagnosis Differential diagnosis	Image + history + imaging findings Image + history + imaging findings	History + imaging findings Image only History + imaging findings Image only	Accuracy **78%** Accuracy 76% Accuracy 32% Accuracy 84% *^,+^ Accuracy 83%* Accuracy 54%**^+^**	* *p* < 0.001 **^+^** *p* = 0.586
Wang X et al., 2025 [28]	Diagnosis of CAD	MMAN (Baseline + ICCA + CDGA, S + R + C)	Baseline (C) Baseline (R) Baseline (S) Baseline + CDGA (S, C)	F1 **0.7285**, Accuracy **0.8362**, AUC **0.879** F1 0.4107, Accuracy 0.7155, AUC 0.7665 F1 0.5167, Accuracy 0.75, AUC 0.7537 F1 0.62, Accuracy 0.7888, AUC 0.826 F1 0.691, Accuracy 0.819, AUC 0.861	C = clinical data R = rest MPI S = stress MPI *p*-value not reported
Xu et al., 2025 [29]	Diagnosis: MDD vs. non-MDD	Audio + visual + text	Visual + text Text Visual Audio	F1 **0.955**, Accuracy **0.952**, AUC **0.978** F1 0.95, Accuracy 0.948, AUC 0.972 F1 0.948, Accuracy 0.945, AUC 0.971 F1 0.748, Accuracy 0.891, AUC 0.775 F1 0.895, Accuracy 0.743, AUC 0.932	*p*-value not reported
Studies comparing performance between multimodal models and unimodal models					
Hooshangnejad et al., 2024 [30]	Tumor nodule detection	EXACT-Net with EHR data	EXACT-Net without EHR data	Accuracy **70%** Accuracy 20%	*p*-value not reported
Li X et al., 2025 [31]	Diagnosis: CAD vs. non-CAD	Clinical + image	Image only Clinical only	**F1 88.35 ± 0.85,** Accuracy **86.33 ± 1.09**, AUC **0.903** *^,+^ F1 81.94 ± 0.15, Accuracy 77.19 ± 0.41, AUC 0.799 * F1 86.80 ± 1.01, Accuracy 84.50 ± 1.33, AUC 0.879 ^+^	* *p* < 0.001 ^+^ *p* = 0.28
Ma et al., 2025 [32]	Diagnosis of ophthalmic diseases	IOMIDS; multimodal models	Unimodal models (text only)	Accuracy **81.1%** ** Accuracy 72.5% **	** Highest accuracy from external validation test *p*-value not reported
Schmidl et al., 2025 [33]	Diagnosis: SCC, oral leukoplakia, and no lesion	Image and clinical history input	Image only Clinical history only	Accuracy **91.1%** Accuracy 35.6% Accuracy 84.4%	Performance shown was from SCC diagnosis group Combined model also achieved higher accuracy compared to unimodal model in leukoplakia and no lesion group *p*-value not reported
Wang Y et al., 2025 [34]	Diagnosis of SPN as benign or malignant	TIC-LUV model (image + clinical)	US diagnosis CT diagnosis	Accuracy **91%** Accuracy 76% Accuracy 75%	Reported performance was from internal test set TIC-LUV model also achieved highest accuracy compared to image model in external test set *p*-value not reported
Zeljkovic et al., 2025 [35]	Most likely diagnosis classification per ECG	ChatGPT-4–ECG with clinical context (multimodal)	ChatGPT-4–ECG without clinical context (unimodal)	Accuracy **45%** Accuracy 19%	*p* < 0.001
Studies conducted without comparator					
Chiesa-Estomba et al., 2025 * [36]	Diagnosis of laryngeal malignant and premalignant diseases	ChatGPT-4o	No comparator	Accuracy 30%	The model proposed correct potential/differential diagnoses in up to 90% of cases
Li J et al., 2023 [37]	Diagnosis of pulmonary diseases	Study model	No comparator	F1 87.7%, Accuracy 87.8%	

AUC, area under the curve; CAD, coronary artery disease; CDGA, clinical data-guided attention; CT, computed tomography; ECG, electrocardiogram; EHR, electronic health record; ICCA, image-correlated cross-attention; IOMIDS, Intelligent Ophthalmic Multimodal Interactive Diagnostic System; MDD, major depressive disorder; MMAN, multi-modality attention network; SCC, squamous cell carcinoma; SPN, subpleural pulmonary nodules; TIC-LUV, time-intensity curve–empowered lung ultrasound video diagnostic; US, ultrasound.

## Data Availability

No new data were created or analyzed in this study. The data supporting this study can be found in the original publication and reports referenced in the citations.

## Supplementary Materials

The following supporting information can be downloaded at: https://www.mdpi.com/article/10.3390/healthcare14131877/s1, Data S1: The PRISMA 2020 checklist; Data S2: Search methodology; Data S3: A detailed quality assessment with the PROBAST + AI tool; Data S4: Study characteristics categorized by medical specialty.
