# Supplementary material for: Do Multimodal Vision-Language Models Enhance the Medical Diagnostic Process? A Systematic Review
Source: Healthcare (Basel). 2026 Jun 26;14(13):1877. doi: 10.3390/healthcare14131877 (PMC13361721; doi:10.3390/healthcare14131877)
Supplement: Supplementary file 1 [file healthcare-14-01877-s001.zip › healthcare-4360275-supplementary.pdf]

Supplemental Materials

Do Multimodal Vision-Language Models Enhance Medical Diagnostic Process? A Systematic Review

Data S1. The PRISMA 2020 checklist

S1.1 The PRISMA 2020 main checklist

Section and Topic	Item #	Checklist item	Location where item is reported
**TITLE**			
Title	1	Identify the report as a systematic review.	Title (page 1, line 1)
**ABSTRACT**			
Abstract	2	See the PRISMA 2020 for Abstracts checklist.	
**INTRODUCTION**			
Rationale	3	Describe the rationale for the review in the context of existing knowledge.	Background (page 3, line 53-74)
Objectives	4	Provide an explicit statement of the objective(s) or question(s) the review addresses.	Background (page 3, line 75-77)
**METHODS**			
Eligibility criteria	5	Specify the inclusion and exclusion criteria for the review and how studies were grouped for the syntheses.	Eligibility criteria (page 4, line 107-120)
Information sources	6	Specify all databases, registers, websites, organisations, reference lists and other sources searched or consulted to identify studies. Specify the date when each source was last searched or consulted.	Search strategy (page 3-4, line 80-105), Supplementary Data 2
Search strategy	7	Present the full search strategies for all databases, registers and websites, including any filters and limits used.	Search strategy (page 3-4, line 80-105), Supplementary Data 2
Selection process	8	Specify the methods used to decide whether a study met the inclusion criteria of the review, including how many reviewers screened each record and each report retrieved, whether they worked independently, and if applicable, details of automation tools used in the process.	Study selection (page 4, line 122-126)
Data collection process	9	Specify the methods used to collect data from reports, including how many reviewers collected data from each report, whether they worked independently, any processes for obtaining or confirming data from study investigators, and if applicable, details of automation tools used in the process.	Data extraction and quality assessment (page 4-5, line 129-135)
Data items	10a	List and define all outcomes for which data were sought. Specify whether all results that were compatible with each outcome domain in each study were sought (e.g. for all measures, time points, analyses), and if not, the methods used to decide which results to collect.	Data extraction and quality assessment (page 4-5, line 129-135)

	10b	List and define all other variables for which data were sought (e.g. participant and intervention characteristics, funding sources). Describe any assumptions made about any missing or unclear information.	Data extraction and quality assessment (page 4-5, line 129-135)
Study risk of bias assessment	11	Specify the methods used to assess risk of bias in the included studies, including details of the tool(s) used, how many reviewers assessed each study and whether they worked independently, and if applicable, details of automation tools used in the process.	Data extraction and quality assessment (page 5, line 136-142)
Effect measures	12	Specify for each outcome the effect measure(s) (e.g. risk ratio, mean difference) used in the synthesis or presentation of results.	Data synthesis and analysis (page 5, line 145-151)
Synthesis methods	13a	Describe the processes used to decide which studies were eligible for each synthesis (e.g. tabulating the study intervention characteristics and comparing against the planned groups for each synthesis (item #5)).	Data synthesis and analysis (page 5, line 145-151)
	13b	Describe any methods required to prepare the data for presentation or synthesis, such as handling of missing summary statistics, or data conversions.	Data synthesis and analysis (page 5, line 145-151)
	13c	Describe any methods used to tabulate or visually display results of individual studies and syntheses.	Data synthesis and analysis (page 5, line 145-151)
	13d	Describe any methods used to synthesize results and provide a rationale for the choice(s). If meta-analysis was performed, describe the model(s), method(s) to identify the presence and extent of statistical heterogeneity, and software package(s) used.	Data synthesis and analysis (page 5, line 145-151)
	13e	Describe any methods used to explore possible causes of heterogeneity among study results (e.g. subgroup analysis, meta-regression).	Data synthesis and analysis (page 5, line 145-151)
	13f	Describe any sensitivity analyses conducted to assess robustness of the synthesized results.	N/A due to meta-analysis was not conducted
Reporting bias assessment	14	Describe any methods used to assess risk of bias due to missing results in a synthesis (arising from reporting biases).	N/A due to meta-analysis was not conducted
Certainty assessment	15	Describe any methods used to assess certainty (or confidence) in the body of evidence for an outcome.	N/A due to meta-analysis was not conducted
**RESULTS**			
Study selection	16a	Describe the results of the search and selection process, from the number of records identified in the search to the number of studies included in the review, ideally using a flow diagram.	Study Characteristics (page 6, line 154-184), Figure 1
	16b	Cite studies that might appear to meet the inclusion criteria, but which were excluded, and explain why they were excluded.	Study Characteristics (page 6, line 154-184), Figure 1
Study characteristics	17	Cite each included study and present its characteristics.	Study Characteristics (page 6, line 163-184), Figure 1
Risk of bias in studies	18	Present assessments of risk of bias for each included study.	Methodological quality (page 8, line 253-262), Table 3

Results of individual studies	19	For all outcomes, present, for each study: (a) summary statistics for each group (where appropriate) and (b) an effect estimate and its precision (e.g. confidence/credible interval), ideally using structured tables or plots.	Table 2
Results of syntheses	20a	For each synthesis, briefly summarise the characteristics and risk of bias among contributing studies.	Results (page 6-8, line 163-262), Table 1, Table 3
	20b	Present results of all statistical syntheses conducted. If meta-analysis was done, present for each the summary estimate and its precision (e.g. confidence/credible interval) and measures of statistical heterogeneity. If comparing groups, describe the direction of the effect.	Results (page 6-8, line 186-233), Table 2
	20c	Present results of all investigations of possible causes of heterogeneity among study results.	N/A due to meta-analysis was not conducted
	20d	Present results of all sensitivity analyses conducted to assess the robustness of the synthesized results.	N/A due to meta-analysis was not conducted
Reporting biases	21	Present assessments of risk of bias due to missing results (arising from reporting biases) for each synthesis assessed.	N/A due to meta-analysis was not conducted
Certainty of evidence	22	Present assessments of certainty (or confidence) in the body of evidence for each outcome assessed.	N/A due to meta-analysis was not conducted
**DISCUSSION**			
Discussion	23a	Provide a general interpretation of the results in the context of other evidence.	Discussion (page 8-11, line 265-383)
	23b	Discuss any limitations of the evidence included in the review.	Discussion (page 8-11, line 265-404)
	23c	Discuss any limitations of the review processes used.	Discussion (page 11, line 396-404)
	23d	Discuss implications of the results for practice, policy, and future research.	Discussion and Conclusions (page 8-12, line 265-416
**OTHER INFORMATION**			
Registration and protocol	24a	Provide registration information for the review, including register name and registration number, or state that the review was not registered.	Search strategy (page 3, line 81-82)
	24b	Indicate where the review protocol can be accessed, or state that a protocol was not prepared.	Search strategy (page 3, line 81-82)
	24c	Describe and explain any amendments to information provided at registration or in the protocol.	Search strategy (page 3, line 81-82)
Support	25	Describe sources of financial or non-financial support for the review, and the role of the funders or sponsors in the review.	Funding (page 12, line 429-430)
Competing interests	26	Declare any competing interests of review authors.	Declaration of competing interests (page 12, line 431-433)
Availability of data, code and other materials	27	Report which of the following are publicly available and where they can be found: template data collection forms; data extracted from included studies; data used for all analyses; analytic code; any other materials used in the review.	Data Availability Statement (page 12, line 434-435)

S1.2 PRISMA 2020 for Abstracts Checklist

Section and Topic	Item #	Checklist item	Reported (Yes/No)
**TITLE**			
Title	1	Identify the report as a systematic review.	Yes
**BACKGROUND**			
Objectives	2	Provide an explicit statement of the main objective(s) or question(s) the review addresses.	Yes
**METHODS**			
Eligibility criteria	3	Specify the inclusion and exclusion criteria for the review.	Yes
Information sources	4	Specify the information sources (e.g. databases, registers) used to identify studies and the date when each was last searched.	Yes
Risk of bias	5	Specify the methods used to assess risk of bias in the included studies.	Yes
Synthesis of results	6	Specify the methods used to present and synthesise results.	Yes
**RESULTS**			
Included studies	7	Give the total number of included studies and participants and summarise relevant characteristics of studies.	Yes
Synthesis of results	8	Present results for main outcomes, preferably indicating the number of included studies and participants for each. If meta-analysis was done, report the summary estimate and confidence/credible interval. If comparing groups, indicate the direction of the effect (i.e. which group is favoured).	Yes
**DISCUSSION**			
Limitations of evidence	9	Provide a brief summary of the limitations of the evidence included in the review (e.g. study risk of bias, inconsistency and imprecision).	Yes
Interpretation	10	Provide a general interpretation of the results and important implications.	Yes
**OTHER**			
Funding	11	Specify the primary source of funding for the review.	Yes
Registration	12	Provide the register name and registration number.	Yes

From: Page MJ, McKenzie JE, Bossuyt PM, Boutron I, Hoffmann TC, Mulrow CD, et al. The PRISMA 2020 statement: an updated guideline for reporting systematic reviews. BMJ 2021;372:n71. doi: 10.1136/bmj.n71. This work is licensed under CC BY 4.0. To view a copy of this license, visit https://creativecommons.org/licenses/by/4.0/

Data S2. Search Methodology

The PICOTS criteria

The Population, Index model, Comparator model, Outcome, Timing, Setting, and intended use of the prediction model (PICOTS) criteria were defined as:

- Population – Adult patients in any hospital setting
- Index model – VLMs utilizing real patient textual history and imaging for diagnosis
- Comparator – Traditional diagnostic models e.g. human or physician, or no intervention
- Outcome – Diagnostic performance, time to diagnosis, or diagnostic errors
- Timing – Any timepoint
- Setting – Any service in a hospital

Development

A Medical librarian (M.M.) developed the search strategy for Embase.

Limits and filters

We applied English language and publication date limits. We applied subject areas in Scopus. We did not apply published search filters. We detail all limits in full search strategies found below.

Validation

The search strategy successfully retrieved all prespecified benchmark studies as listed here:

https://doi.org/10.1038/s41591-023-02504-3
https://doi.org/10.1038/s41591-024-02856-4
https://doi.org/10.1038/s41591-024-02959-y
https://doi.org/10.1007/s00371-024-03579-w

Peer review

The authors thank Dana Gerberi, MLIS, AHIP from the Mayo Clinic Libraries in Rochester, MN for peer review of the Embase database search strategy.

Database searches

Embase 1974 to 2025 December 15 (Wolters Kluwer Ovid interface)

Date searched: December 17, 2025

Records retrieved: 2993

Language: limited to English

Publication dates: limited to records published from 2000 – 2025

(((vision OR visual) ADJ (language*)).ti,ab,kf,dq. AND (AI OR artificial-intelligence OR computer-vision OR machine-learning OR multi-modal OR multimodal OR (multiple ADJ modalit*) OR neural-

network*).ti,ab,kf.) OR ((((VLM OR VLMs OR VQA OR VQAs) AND (vision OR visual)) OR ((vision OR visual) ADJ3 (language OR language-foundation OR question* OR understand* OR recogni*) ADJ3 (model OR models)) OR ((text*) ADJ (to$1) ADJ (image*)) OR ((text*) ADJ (to$1) ADJ (video*)) OR ((image*) ADJ (to$1) ADJ (text*)) OR ((image*) ADJ (to$1) ADJ (video*))).ti,ab,kf,dq.) OR (language model/ OR exp large language model/ OR natural language processing/ AND (image* OR photo* OR picture* OR video*).ti,kf.) OR ((exp artificial intelligence/ OR exp computer vision/ OR exp machine learning/) AND ((image* OR photo* OR picture* OR video*) ADJ7 (caption* OR language* OR text OR texts OR textual)).ti,kf.) OR (image analysis/ OR exp image processing/ OR photography/ OR exp medical photography/ OR exp videorecording/ AND ((image* OR photo* OR picture* OR video*) ADJ7 (caption* OR ((large OR natural) ADJ (language*)) OR LLM OR LLMs OR NLP OR NLPS OR text OR texts OR textual)).ti,kf.) OR (((image OR photo* OR picture* OR video*) ADJ (analys#s OR caption* OR interpret* OR process* OR recogni*) ADJ (model OR models))).ti,ab,kf,dq. AND english.la AND 2000:2025.(sa_year)

Ovid MEDLINE(R) 1946 to Present and Epub Ahead of Print, In-Process & Other Non-Indexed Citations and Ovid MEDLINE(R) Daily

Date searched: December 17, 2025

Records retrieved: 1995

Language: limited to English

Publication dates: limited to records published from 2000 – 2025

(((vision OR visual) ADJ (language*)).ti,ab,kf. AND (AI OR artificial-intelligence OR computer-vision OR machine-learning OR multi-modal OR multimodal OR (multiple ADJ modalit*) OR neural-network*).ti,ab,kf.) OR ((((VLM OR VLMs OR VQA OR VQAs) AND (vision OR visual)) OR ((vision OR visual) ADJ3 (language OR language-foundation OR question* OR understand* OR recogni*) ADJ3 (model*)) OR ((text*) ADJ (to$1) ADJ (image*)) OR ((text*) ADJ (to$1) ADJ (video*)) OR ((image*) ADJ (to$1) ADJ (text*)) OR ((image*) ADJ (to$1) ADJ (video*))).ti,ab,kf.) OR (natural language processing/ AND (image* OR photo* OR picture* OR video*).ti,kf.) OR ((exp artificial intelligence/ OR exp machine learning/) AND ((image* OR photo* OR picture* OR video*) ADJ7 (caption* OR language* OR text OR texts OR textual)).ti,kf.) OR (Image interpretation, computer-assisted/ OR image processing, computer-assisted/ OR Pattern recognition, automated/ OR exp photography/ OR exp video recording/ AND ((image* OR photo* OR picture* OR video*) ADJ7 (caption* OR ((large OR natural) ADJ (language*)) OR LLM OR LLMs OR NLP OR NLPS OR text OR texts OR textual)).ti,kf.) OR (((image OR photo* OR picture* OR video*) ADJ (analys#s OR caption* OR interpret* OR process* OR recogni*) ADJ (model OR models))).ti,ab,kf. AND english.la AND 2000:2025.(sa_year)

EBM Reviews - Cochrane Central Register of Controlled Trials November 2025 (Wolters Kluwer Ovid interface)

Date searched: December 17, 2025

Records retrieved: 48

Language: limited to English

Publication dates: limited to records published from 2000 – 2025

(((vision OR visual) ADJ (language*)).ti,ab,kf. AND (AI OR artificial-intelligence OR computer-vision OR machine-learning OR multi-modal OR multimodal OR (multiple ADJ modalit*) OR neural-network*).ti,ab,kw,sh.) OR ((((VLM OR VLMs OR VQA OR VQAs) AND (vision OR visual)) OR ((vision OR visual) ADJ3 (language OR language-foundation OR question* OR understand* OR recogni*) ADJ3 (model*)) OR ((text*) ADJ (to$1) ADJ (image*)) OR ((text*) ADJ (to$1) ADJ (video*)) OR ((image*) ADJ (to$1) ADJ (text*)) OR ((image*) ADJ (to$1) ADJ (video*))).ti,ab,kw,sh.) OR (natural language processing/ AND (image* OR photo* OR picture* OR video*).ti,kw,sh.) OR ((exp artificial intelligence/ OR exp machine learning/) AND ((image* OR photo* OR picture* OR video*) ADJ7 (caption* OR language* OR text OR texts OR textual)).ti,kw,sh.) OR (Image interpretation, computer-assisted/ OR image processing, computer-assisted/ OR Pattern recognition, automated/ OR exp photography/ OR exp video recording/ AND ((image* OR photo* OR picture* OR video*) ADJ7 (caption* OR ((large OR natural) ADJ (language*)) OR LLM OR LLMs OR NLP OR NLPS OR text OR texts OR textual)).ti,kw,sh.) OR (((image OR photo* OR picture* OR video*) ADJ (analys#s OR caption* OR interpret* OR process* OR recogni*) ADJ (model OR models))).ti,ab,kw,sh. AND english.la AND 2000:2025.(sa_year)

Scopus*

Date searched: December 17, 2025

Records retrieved: 3106

Language: limited to English

Subject Areas: limited to Medicine, Neuroscience, and Health Professions

Publication dates: limited to records published from 2000 - 2025

TITLE-ABS-KEY (((((vision OR visual) PRE/0 (language*))) AND (AI OR artificial-intelligence OR computer-vision OR machine-learning OR multi-modal OR multimodal OR (multiple PRE/0 modalit*) OR neural-network*)) OR (((VLM OR VLMs OR VQA OR VQAs) AND (vision OR visual)) OR ((vision OR visual) W/2 (language OR language-foundation OR question* OR understand* OR recogni*) W/2 (model*)) OR (text-to-image OR text-to-video OR image-to-text OR video-to-text)) OR ((image* OR photo* OR picture* OR video*) W/6 (caption* OR ((large OR natural) W/0 (language*)) OR LLM OR LLMs OR NLP OR NLPS OR text OR texts OR textual) W/6 (model OR models)) OR (((image OR photo* OR picture* OR video*) PRE/0 (analys#s OR caption* OR interpret* OR process* OR recogni*) PRE/0 (model OR models)))) AND PUBYEAR > 1999 AND PUBYEAR < 2026 AND (LIMIT-TO (LANGUAGE, "English")) AND (LIMIT-TO (SUBJAREA, "MEDI") OR LIMIT-TO (SUBJAREA, "NEUR") OR LIMIT-TO (SUBJAREA, "HEAL"))

*Scopus search retrieved 3130 records but only 3106 exported to EndNote. This is a known, inconsistent but universally occurring issue with Scopus export function. It is impossible to determine which of the 124 records in the results set did not export.

Science Citation Index Expanded (SCI-Expanded)—1975-present and Emerging Sources Citation Index (ESCI)—2018-present (Web of Science)

Date searched: December 17, 2025

Records retrieved: 757

Language: limited to English

TS= (((((vision OR visual) NEAR/0 (language*))) AND (AI OR artificial-intelligence OR computer-vision OR machine-learning OR multi-modal OR multimodal OR (multiple NEAR/0 modalit*) OR neural-network*)) OR (((VLM OR VLMs OR VQA OR VQAs) AND (vision OR visual)) OR ((vision OR visual) NEAR/2 (language OR language-foundation OR question* OR understand* OR recogni*) NEAR/2 (model*)) OR (text-to-image OR text-to-video OR image-to-text OR video-to-text)) OR ((image* OR photo* OR picture* OR video*) NEAR/6 (caption* OR ((large OR natural) NEAR/0 (language*)) OR LLM OR LLMs OR NLP OR NLPS OR text OR texts OR textual) NEAR/6 (model OR models)) OR ((image OR photo* OR picture* OR video*) NEAR/0 (analysis OR caption* OR interpret* OR process* OR recogni*) NEAR/0 (model OR models))) AND (TI=(clinic* OR diagnos* OR disease OR ECG OR echo* OR hospital OR microbiology OR pathology OR patient OR radiology OR medical OR medicine) OR AK=(diagnos* OR ECG OR echo* OR microbiology OR pathology OR radiology) OR TS=((medical OR medicine) NEAR/5 (image OR imaging)))

Preprint Citation Index (PCI)—1991-present (Web of Science)

Date searched: December 17, 2025

Records retrieved: 840

TS= (((((vision OR visual) NEAR/0 (language*))) AND (AI OR artificial-intelligence OR computer-vision OR machine-learning OR multi-modal OR multimodal OR (multiple NEAR/0 modalit*) OR neural-network*)) OR (((VLM OR VLMs OR VQA OR VQAs) AND (vision OR visual)) OR ((vision OR visual) NEAR/2 (language OR language-foundation OR question* OR understand* OR recogni*) NEAR/2 (model*)) OR (text-to-image OR text-to-video OR image-to-text OR video-to-text)) OR ((image* OR photo* OR picture* OR video*) NEAR/6 (caption* OR ((large OR natural) NEAR/0 (language*)) OR LLM OR LLMs OR NLP OR NLPS OR text OR texts OR textual) NEAR/6 (model OR models)) OR ((image OR photo* OR picture* OR video*) NEAR/0 (analysis OR caption* OR interpret* OR process* OR recogni*) NEAR/0 (model OR models))) AND (TI=(clinic* OR diagnos* OR disease OR ECG OR echo* OR hospital OR microbiology OR pathology OR patient OR radiology OR medical OR medicine) OR AK=(diagnos* OR ECG OR echo* OR microbiology OR pathology OR radiology) OR TS=((medical OR medicine) NEAR/5 (image OR imaging)))

IEEE Xplore

Date searched: December 17, 2025

Records retrieved: 476

Publication dates: limited to records published from 2000 – 2025

((("All Metadata":"visual" ONEAR/1 "language") OR ("All Metadata":"vision" ONEAR/1 "language")) AND (("All Metadata":"clinic*" OR "diagnos*" OR "disease*" OR "ECG" OR "echo*" OR "hospital*" OR "microbiolog*" OR "patholog*" OR "patient*" OR "radiolog*" OR "medical" OR "medicine")))

Data S3. A detailed quality assessment by PROBAST+AI tool

S3.1 Model development

Study	Participants		Predictors		Outcome		Analysis	Overall judgement of model development	
	Quality concern	Applicability concern	Quality concern	Applicability concern	Quality concern	Applicability concern	Quality concern	Quality concern	Applicability concern
Karaman et al., 2026 [10]									
Sorin et al., 2025 [8]									
Tanyeri et al., 2025 [24]									
Wu et al., 2025 [25]	Low concern	Low concern	Low concern	Low concern	Low concern	Low concern	Low concern	Low	Low
Legrain et al., 2025 [26]									
Oikonomou et al., 2026 [11]	Unclear	Low concern	Unclear	Low concern	Low concern	Low concern	Unclear	Unsure	Low
Schramm et al., 2025 [27]									
Sun et al., 2026 [9]									
Wang X et al., 2025 [28]	High concern	Low concern	High concern	Low concern	Low concern	Low concern	Unclear	High	Low
Xu et al., 2025 [29]	Low concern	High concern	Low concern	Low concern	Low concern	Low concern	Unclear	Unsure	Low
Hooshangnejad et al., 2024 [30]	High concern	High concern	Unclear	Low concern	High concern	Low concern	High concern	High	High
Li X et al., 2025 [31]	Low concern	Low concern	Low concern	Low concern	Low concern	High concern	Low concern	High	High
Ma et al., 2025 [32]	Unclear	Low concern	Low concern	Low concern	Low concern	Low concern	High concern	High	Low
Schmidl et al., 2025 [33]									
Wang Y et al., 2025 [34]	Low concern	Low concern	Low concern	Low concern	Low concern	Low concern	Unclear	Unsure	Low

Zeljkovic et al., 2025 [35]									
Chiesa-Estomba et al., 2025 [36]									
Li J et al., 2023 [37]	High concern	High concern	High concern	High concern	Unclear	Unclear	High concern	High	High

S3.2 Model evaluation

Study	Participants		Predictors		Outcome		Analysis	Overall judgement of model evaluation	
	Quality concern	Applicability concern	Quality concern	Applicability concern	Quality concern	Applicability concern	Quality concern	Quality concern	Applicability concern
Karaman et al., 2026 [10]	High concern	High concern	Low concern	Low concern	Unclear	Low concern	High concern	High	High
Sorin et al., 2025 [8]	High concern	Unclear	Unclear	Unclear	Low concern	Low concern	High concern	High	Unsure
Tanyeri et al., 2025 [24]	High concern	Unclear	Low concern	Unclear	Low concern	Low concern	High concern	High	Unsure
Wu et al., 2025 [25]	Low concern	Low concern	Low concern	Low concern	Low concern	Low concern	Low concern	Low	Low
Legrain et al., 2025 [26]	High concern	Low concern	High concern	Low concern	Unclear	Low concern	High concern	High	Low
Oikonomou et al., 2026 [11]	Unclear	Low concern	Unclear	Low concern	Low concern	Low concern	Unclear	Unsure	Low
Schramm et al., 2025 [27]	High concern	High concern	High concern	High concern	Low concern	High concern	High concern	High	High
Sun et al., 2026 [9]	High concern	Low concern	Unclear	Unclear	Low concern	Low concern	High concern	High	Unsure
Wang X et al., 2025 [28]	High concern	Low concern	High concern	Low concern	Low concern	Low concern	Unclear	High	Low
Xu et al., 2025 [29]	Unclear	High concern	Low concern	Low concern	Low concern	Low concern	Unclear	Unsure	Low

Hooshangnejad et al., 2024 [30]	High concern	High concern	High concern	Low concern	High concern	Low concern	High concern	High	High
Li X et al., 2025 [31]	Low concern	Low concern	Low concern	Low concern	High concern	High concern	Low concern	Low	High
Ma et al., 2025 [32]	Unclear	Low concern	Low concern	Low concern	Low concern	Low concern	High concern	High	Low
Schmidl et al., 2025 [33]	High concern	Low concern	Unclear	Unclear	Low concern	Low concern	High concern	High	Unsure
Wang Y et al., 2025 [34]	Low concern	Low concern	Low concern	Low concern	Low concern	Low concern	Low concern	Low	Low
Zeljkovic et al., 2025 [35]	High concern	Low concern	Low concern	Low concern	Low concern	Low concern	Unclear	High	Low
Chiesa-Estomba et al., 2025 [36]	High concern	Unclear	Unclear	Unclear	Low concern	Low concern	High concern	High	Unsure
Li J et al., 2023 [37]	High concern	High concern	High concern	High concern	Unclear	Unclear	High concern	High	High

Data S4. Study characteristics categorized by medical specialty

Specialty	Study	Purpose of the study	Population	Datasets	Intervention	Comparator
Cardiovascular diseases	Li X et al., 2025 [31]	To develop a DL-based visual and multimodal detection framework for CAD using retinal images	Patients who underwent successful CAG	383 patients<br><br>Text: 45 clinical indicators<br>Image: OCT/OCTA	**Multimodal VLMs**	Unimodal models
	Oikonomou et al., 2026 [11]	To develop multimodal models combining EHR and ECG data for screening in health systems	Training: patients who underwent ECG and TTE<br>Validation: patients who underwent ECG and CMR	Training: 159,322 patients<br>Internal validation: 8,979<br>External validation: 38,749 from 2 external datasets<br><br>Text: EHR data<br>Image: ECG and TTE	**TARGET-AI** with targeted strategy (EHR then AI-ECG)	- TARGET-AI with untargeted strategy (AI-ECG + EHR)<br>- TARGET-AI with untargeted strategy (AI-ECG alone)
	Wang X et al., 2025 [28]	To develop a multi-modality attention network integrating MPI and clinical data for CAD evaluation.	Patients who underwent MPI scans	1468 patients<br><br>Text: clinical variables<br>Image: 1,072 MPI	**Multimodal VLMs**	Unimodal models
	Zeljkovic et al., 2025 [35]	Evaluate GPT-4's ability to interpret 12-lead ECGs with and without clinical context.	Adults in hospital setting from CaRD registry; 150 12-lead ECGs	150 cases<br><br>Text: clinical scenario<br>Image: 150 ECG images	**ChatGPT-4 with multimodal input**	ChatGPT-4 with unimodal input
Radiology	Karaman et al., 2026 [10]	To assess the diagnostic accuracy of 3 LLMs—ChatGPT-4o, Grok-3, and Claude 3.7 Sonnet—for schwannoma and meningioma	Patients with vestibular schwannoma or meningioma located in the CPA	53 patients<br><br>Text: clinical data<br>Image: 53 pairs of selected slices from CT and MRI	**- ChatGPT-4o**<br>**- Grok-3**<br>**- Claude 3.7 Sonnet**	- Resident radiologist<br>- Experienced neuroradiologist
	Schramm et al., 2025 [27]	To evaluate the impact of varying multimodal input elements on the accuracy of GPT-4V–based brain MRI differential diagnosis.	Patients with brain MRI images	60 patients selected by physicians<br><br>Text: brief medical history, image description<br>Image: 60 MRI	**ChatGPT-4V with multimodal input**	ChatGPT-4V with unimodal input
	Sun et al., 2026 [9]	To evaluate the diagnostic performance of GPT-4o in interpreting radiological images, imaging findings, and patient histories for brain tumor diagnosis	Pre-operative brain tumor patients	239 patients<br><br>Text: brief clinical history<br>Image: MRI slice	**ChatGPT-4o with multimodal input**	ChatGPT-4o with unimodal input

Specialty	Study	Purpose of the study	Population	Datasets	Intervention	Comparator
Ophthalmology	Ma et al., 2025 [32]	To develop models for diagnosing ophthalmic diseases.	Patients with ophthalmic diseases	Development: 90 cases<br>Internal validation: 2292<br>External validation: 2940<br><br>Text: real dialogs with clinical data<br>Image: slit-lamp and smartphone-acquired image	**IOMIDS**; Multimodal models	Unimodal models
	Sorin et al., 2025 [8]	To evaluate the performance of GPT-4V in an integrated analysis of ocular images and clinical text	Patients with ophthalmologic conditions	40 patients selected by physicians<br><br>Text: selected patient information e.g. clinical contexts<br>Image: patient images	**ChatGPT-4V**	- GPT-4V without clinical context<br>- Non-ophthalmologic physician 1<br>- Non-ophthalmologic physician 2
	Wu et al., 2025 [25]	To develop a model as a copilot for ophthalmology	Patients with ophthalmic diseases	Pretraining:<br>- 14.5 million images in 5 modalities: CFP, OCT, UWF, FFA, and external eye photo<br>- 0.4 million clinical texts<br>Test - RCT in Experiment 3: 668 patients<br><br>Text: clinical text<br>Image: CFP, OCT, UWF, FFA, and external eye photo	**EyeFM + Physician**	Physician alone
Otolaryngology	Chiesa-Estomba et al., 2025 [36]	To evaluate ChatGPT-4o in analyzing clinical fiberoptic videos of suspected laryngeal malignancies compared to expert clinicians	Patients consulting for primary laryngeal disease	20 patients<br><br>Text: clinical history<br>Image: CT and laryngeal fiberoptic video examinations	**ChatGPT-4o**	No comparator
	Legrain et al., 2025* [26]	To investigate the performance of 3 LLMs as adjunctive tools in analyzing clinical pictures	Patients consulting at for primary laryngeal symptoms	50 patients<br>Text: clinical data	**ChatGPT-4o**	- DeepSeek<br>- Claude-3.7-Sonnet

Specialty	Study	Purpose of the study	Population	Datasets	Intervention	Comparator
		of common phoniatric disorders		Image: laryngostroboscopic images		
	Schmidl et al., 2025 [33]	To assess LLMs ability to differentiate between SCC, premalignant lesions, and benign or lesion-free conditions	Patients with or without oral lesion	45 patients<br>Text: clinical data<br>Image: 45 lesion images	**ChatGPT with multimodal input**	ChatGPT with unimodal input
Pulmonary diseases	Hooshangnejad et al., 2024 [30]	To develop a novel EHR-guided tumor detection via auto-segmentation method	Training and validation: LIDC-IDRI public data with 201 CT<br><br>Test: Patients with lung tumor	Tested on 10 patient EHR data from hospital database<br><br>Text: EHR data<br>Image: MRI	**EXACT-Net with EHR data**	EXACT-Net without EHR data
	Li J et al., 2023 [37]	To develop an interpretable AI framework for diagnosing pulmonary diseases using multimodal data	Patients with lung disease	EHR data from 1000 patients<br><br>Text: 1,000 EHR data from hospital database<br>Image: 1000 X-rays	**Multimodal VLMs**	No comparator
	Wang Y et al., 2025 [34]	To develop a DL model based on DMUS video for the differential diagnosis of benign and malignant SPNs.	Participants with subpleural pulmonary nodules	372 participants<br>Training: 154<br>Validation: 39<br>Internal Test: 88<br>External Test: 91<br><br>Text: clinical information<br>Image: US video	**Multimodal VLMs**	Unimodal models
Emergency medicine	Tanyeri et al., 2025 [24]	To evaluate the diagnostic performance of GPT-4o in emergency abdominal CT cases compared to radiology residents with varying levels of experience	Patients in emergency department	45 patients<br><br>Text: summarized clinical description<br>Image: CT	**ChatGPT-4o**	- Advanced-experienced resident group<br>- Intermediate-experienced resident group<br>- Limited-experienced resident group
Psychiatry	Xu et al., 2025 [29]	To develop an automated multimodal AI model	MDD patients and healthy volunteers	Training and validation: 289 patients	**Multimodal VLMs**	Unimodal models

Specialty	Study	Purpose of the study	Population	Datasets	Intervention	Comparator
		(text/audio/video) for depression detection		External validation: 100 patients<br><br>Text, audio, and video data are from interview video		

AI, artificial intelligence; CAD, coronary artery disease; CAG, Coronary angiography; CaRD, Cardiology Research Dubrava registry; CDGA, clinical data-guided attention; CFP, color fundus photography; CMR, cardiac magnetic resonance imaging; CPA, cerebellopontine angle; CT, computed tomography; DL, deep learning; DMUS, dual-modality ultrasound; ECG, electrocardiogram; EHR, electronic health record; FFA, fundus fluorescein angiography; IOMIDS, Intelligent Ophthalmic Multimodal Interactive Diagnostic System; LIDC-IDRI, Lung Image Database Consortium imaging collection; LLM, large language model; MPI, myocardial perfusion imaging; MRI, magnetic resonance imaging; OCT, optical coherence tomography; OCTA, optical coherence tomography angiography; RCT, randomized controlled trial; SCC, squamous cell carcinoma SPN, subpleural pulmonary nodules; TTE, transthoracic echocardiogram; US, ultrasound; UWF, ultra-widefield imaging; VLM, vision-language model
